# Studying the heterogeneous pathogenesis of canine diabetes: Observational characterization of an island population

**DOI:** 10.1002/vms3.452

**Published:** 2021-02-23

**Authors:** Yeray Brito‐Casillas, Carlos Melián, Angela Holder, Julia C Wiebe, Ana Navarro, Óscar Quesada‐Canales, Ana B Expósito‐Montesdeoca, Brian Catchpole, Ana M Wägner

**Affiliations:** ^1^ Instituto Universitario de Investigaciones Biomédicas y Sanitarias Universidad de Las Palmas de Gran Canaria (ULPGC) Las Palmas de Gran Canaria Spain; ^2^ Servicio de Endocrinología y Nutrición Complejo Hospitalario Universitario Insular Materno Infantil de Gran Canaria Las Palmas de Gran Canaria Spain; ^3^ Departamento de Patología Animal Producción Animal Bromatología y Tecnología de los Alimentos ULPGC Arucas Spain; ^4^ Department of Pathology & Pathogen Biology Royal Veterinary College University of London London UK; ^5^ Grupo de Investigación en Acuicultura (GIA) ULPGC Arucas Spain; ^6^ Unidad de Histología y Patología Veterinaria Instituto Universitario de Sanidad Animal (IUSA) ULPGC Arucas Canarias Spain

**Keywords:** autoimmune diabetes, diabetes secondary to dioestrus, pancreas, spontaneous diabetes

## Abstract

**Background:**

Canine diabetes mellitus has mostly been studied in northern European, Australian and American populations, whereas other regions have received less attention.

**Objectives:**

We evaluated the epidemiological, clinical and histopathological features of diabetic dogs in Gran Canaria, Spain.

**Methods:**

Prevalence and incidence were estimated. Clinical features were analysed, and serum and genomic DNA were obtained. Dogs with presumed idiopathic or immune‐mediated diabetes, were DLA‐typed and antibodies against GAD65 and IA‐2 were assessed. Pancreases from ten diabetic dogs were examined and compared with pancreases from non‐diabetic dogs.

**Results and conclusions:**

Twenty‐nine diabetic dogs were identified in a population of 5,213 (prevalence: 0.56%; incidence: 0.37%). Most were female (79%) and sexually intact (87% of females, 83% of males). Diabetes secondary to dioestrus (55.2%) and insulin‐deficient diabetes (20.7%) were the most frequent types. Antibodies against GAD65 and IA‐2 were identified in two out of five cases and DLA‐genotyping revealed novel haplotypes. Breed distribution differed between diabetic and non‐diabetic dogs. Reduced number of pancreatic islets and β‐cell mass were observed, with vacuolation of islet cells and ductal epithelium. In this population, where neutering is not standard practice, diabetes secondary to dioestrus is the most frequent diabetes subtype. Genetic susceptibility also differed from previous studies. These results support the heterogeneous pathogenesis of canine diabetes.

## Graphical abstract

After evaluating the epidemiological, clinical and histopathological features of diabetic dogs from the Canary Islands, in this population the most common type of diabetes is dioestrus diabetes, and the breed‐specific risk of diabetes differed from previous studies. These results, obtained in a relatively small population, support the heterogeneous pathogenesis of cDM.

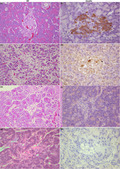

## 1 BACKGROUND

Canine diabetes mellitus (cDM) has been proposed as a spontaneous animal model of human latent autoimmune diabetes of the adult (LADA), a form of type 1 diabetes (T1D) (Catchpole et al., 2005; O’Kell et al., 2017). However, more studies are still necessary to charaterize this possible model of human T1D, given its heterogeneous pathogenesis (O’Kell et al., 2017). Because of the shared habitat and lifestyle of humans and their companion animals, comparative research on gene–environment interaction could be of particular interest (Delicano et al., 2020; Pöppl et al., 2017). Previous epidemiological studies of cDM have mostly been undertaken in northern European, Australian, Canadian and US dog populations (Ahlgren et al., 2014; Davison et al., 2005; Fall et al., 2007; Fracassi et al., 2004; Guptill et al., 2003; Mattin et al., 2014; Shields et al., 2015; Yoon et al., 2020), with similarities and some differences in terms of incidence and breed, age and sex distributions. Alternative geographical locations have received less attention, but may add valuable information to the understanding of the pathogenesis of cDM.

Controversy exists regarding the involvement of autoimmunity in the pathogenesis of the disease, comparing different geographical regions (Ahlgren et al., 2014). Discrepancies about the causes and classification of cDM have been recently and thoroughly discussed by other authors (O’Kell et al., 2017; Yoon et al., 2020). In this sense, cDM studies in different populations could be of interest to improve our knowledge of this disease.

Although the pathogenesis of pancreatic dysfunction leading to cDM is still poorly understood, β‐cell loss associated with insulin deficiency appears to be the main underlying mechanism of disease (Catchpole et al., 2005; Shields et al., 2015). Histopathological descriptions of pancreases from cDM‐affected dogs reflect a heterogeneous process, including degenerative changes in pancreatic islets with vacuolation, although insulitis with lymphocytic infiltration and generalized pancreatic inflammation have also been reported (Ahlgren et al., 2014; Alejandro et al., 1988; Atkins et al., 1979, 1988; Gepts, 1965; Gepts & Toussaint, 1967; Gilor et al., 2016; Jouvion et al., 2006; O’Kell et al., 2017).

For all these reasons and due to the heterogeneity reported by published studies, some authors highlight the need for additional evaluations of cDM (O’Kell et al., 2017)

We report the results of an observational (longitudinal) study aimed to investigate the epidemiological, clinical and histopathological features of a diabetic dog population from the Canary Islands, with particular focus on immune‐mediated disease, given its potential application as a model for human autoimmune diabetes (Brito‐Casillas et al., 2016; Catchpole et al., 2005; Gale, 2005; O’Kell et al., 2017).

### 1.1 METHODS

Diabetic dogs presented to the Veterinary Teaching Hospital of the University of Las Palmas de Gran Canaria (ULPGC), from January 2009 to January 2012, were included. Disease prevalence was calculated as the number of cDM cases divided by the total number of dogs attending the hospital during the given period, and incidence as the number of newly diagnosed cases divided by the total number of dogs seen per year. Features including age, breed, sex, neutering state, time of diagnosis and clinical signs were recorded. Diabetes was diagnosed based on clinical signs (polyuria, polydipsia and weight loss), hyperglycaemia and glucosuria. Classification of cDM was attempted in each case, in order to provide the most adequate treatment (e.g., neutering in diabetes secondary to dioestrus) (Brito‐Casillas et al., 2016), based on the presumed pathogenesis, as proposed by Catchpole *et al* (Catchpole et al., 2005). Clinical records of previously diagnosed dogs were also reviewed to confirm diagnosis and type of diabetes.

Since sex and neutering state were not available for the whole population, from the 14,513 dogs included in the hospital's clinical records, a random, computer‐based, list was generated, in order to contact dog‐owners and obtain information about neutering. The dogs' owners who were available were consecutively interviewed until 100 dogs were reached.

Blood samples were drawn into EDTA containing tubes and tubes without additives from 26 of the 29 diabetic dogs. Serum was separated and genomic DNA was extracted (GenElute Blood Genomic DNA Kit Miniprep, Sigma‐Aldrich). Anti‐insulin antibodies were assessed by ELISA in 19 insulin‐treated dogs, as described previously (Davison et al., 2003). Diabetic dogs without a history of pancreatitis, hyperadrenocorticism, recent dioestrus, iatrogenic or any other known cause of insulin resistance were considered to have idiopathic or immune‐mediated diabetes (Catchpole et al., 2005). In this group, dog leukocyte antigen (DLA) loci were genotyped, as previously reported by Kennedy et al. (2006). Gene alignment and allele assignment were performed using SBT Engine Software version 2.17 (GenDex). Serological analyses were also performed in this small group. Reactivity to canine glutamate decarboxylase 65 kDa (GAD65) and canine anti‐tyrosine phosphatase/insulinoma antigen‐2 (IA‐2) were measured with an established radio‐immuno‐precipitation assay (RIA), as described previously (Davison et al., 2008). Human antibody positive and negative sera were included as controls.

Pancreases were available from diabetic and non‐diabetic dogs of different breeds, naturally deceased or following humane euthanasia during hospitalization. All pancreases were extracted immediately after death (within 90 min). Tissue samples were fixed in 10% neutral buffered formalin and embedded in paraffin. Sections (5 μm) were mounted on glass slides (Superfrost Plus, Thermo Scientific,), stained with haematoxylin‐eosin and assessed by the Veterinary Pathology Service of the ULPGC (OQC) blinded to diabetes status of the dogs from which the samples were obtained. Insulin content was evaluated by immunohistochemistry, after permeabilization with Triton X‐100 0.05% in PBS, with primary rabbit anti‐insulin antibody (9,168, Santa Cruz Biotechnology) and secondary biotinylated goat‐anti‐rabbit antibody (B2770, Thermofisher Scientific). Images were acquired on an Olympus microscope (BX51, Olympus).

Descriptive statistical analysis was performed. Continuous variables are described as mean (*SD*) or median (range), depending on their (Gaussian or non‐Gaussian) distribution and qualitative variables, as percentages. The distribution of the number of cases diagnosed by month and season were recorded and analysed for seasonality at diagnosis. Odds‐ratios (OR) (95% Confidence Intervals) were calculated and chi‐squared analysis was performed to compare breed frequencies between the diabetic and the non‐diabetic groups. A two‐tailed *p* below .05 was considered significant. Statistical analyses were performed using Microsoft Excel 2011, 14.2.2 (Microsoft Corporation) and IBM SPSS Statistics Version 20 (SPSS Inc.).

### 1.2 RESULTS

From 2009 to 2012, a total of 29 dogs with cDM were identified from a mean total population of 1,738 (±146) dogs per year [mean prevalence 0.56% (0.20) and mean incidence per year 0.37% (0.16)] (see Table 1). Median age at diagnosis was 9.5 years (range 3.1–14 years) and most diabetic dogs were female (79.3%) and not neutered (87% of the females; 83% of the males). Only limited information was available on body weight and body condition scores, but most of the dogs (69%) presented with weight loss prior to their admission. The information obtained from the owners of 100 consecutive, non‐diabetic dogs revealed that 60% were female (*p* =.046 versus the group with cDM). Non‐neutered animals represented 37% of females and 45% of males, which was significantly lower than in the diabetic dogs (*p* <.001 and *p* =.015, respectively). Eleven breeds were represented in the cDM population, compared with 106 breeds identified in the non‐diabetic hospital population (see Table 2). The miniature poodle was the most prevalent breed among dogs with cDM, though fox terrier, dachshund, English cocker spaniel, West Highland white terrier and Andalusian wine‐cellar rat‐hunting dog were also at risk for diabetes. Some breeds seemed to be less prone to developing diabetes, such as the local breeds Presa Canario, Podenco Canario, Bardino Majorero and Pastor Garafiano (Tables 1 and 2).

**TABLE 1:** Number of dogs seen at the hospital and distribution of diabetes between 2009 and 2011

Year	Total number of dogs	Number of diabetic dogs	Newly diagnosed diabetic dogs	Prevalence (%)	Incidence (%)
2009	1747	11	4	0.63	0.23
2010	1587	5	5	0.32	0.32
2011	1879	13	10	0.69	0.54
Total/Annual Mean (*SD*)	5213/1738 (146.2)	29/9.7 (4.2)	19/6.3 (3.2)	0.56 (0.20)	0.40/0.37 (0.16)

**TABLE 2:** Breed distribution among diabetic and non‐diabetic dogs

Breed	Diabetic *N* (%)	Non‐diabetic *N* (%)	Comparison to cross‐breed as reference			Comparison to whole population as reference		
			OR	95% CI	*p* (*Χ* ^2^)	OR	95% CI	*p* (*Χ* ^2^ **)**
Poodle^a^	6 (20.69%)	191 (3.7%)	11.42	3.19 – 40.83	<.01*	6.82	2.74–16.94	<.01*
Cocker Spaniel^b^	4 (13.79%)	153 (2.9%)	9.50	2.35–38.38	<.01*	5.26	1.81–15.30	<.01*
**Cross‐Breed**	**4 (13.79%)**	**1,454 (28.0%)**	**1**	**Reference**	**Reference**	0.41	0.14–1.18	.09
German Shepherd^b^	3 (10.34%)	202 (3.9%)	5.40	1.200–24.29	.01*	2.85	0.85–9.48	.07
Fox Terrier^a^	2 (6.90%)	29 (0.6%)	25.07	4.41–142.36	<.01*	13.17	2.99–57.96	<.01*
Andalusian Wine‐Cellar Rat‐Hunting	2 (6.90%)	91 (1.8%)	7.99	1.44–44.19	<.01*	4.15	0.97–17.69	.04*
Dachshund^a^	2 (6.90%)	34 (0.7%)	21.38	3.79–120.74	<.001*	11.22	2.57–49.06	<.01^a^
West Highland White Terrier^a^	2 (6.90%)	59 (1.1%)	12.32	2.21–68.62	<.01*	6.43	1.50–27.68	<.01*
Yorkshire Terrier^a^	2 (6.90%)	498 (9.6%)	1.46	0.27–7.99	.66	0.70	0.16–2.94	.62
Griffon	1 (3.45%)	37 (0.7%)	9.82	1.07–90.05	.01*	4.97	0.66–37.48	.08
Siberian Husky^a^	1 (3.45%)	49 (0.9%)	7.42	0.81–67.60	.04*	3.74	0.50–28.06	.17

Rest of the breeds present in the population			
French Bull dog	0	398 (7.7%)	OR = 0; CI (0.992–1); *p* >.25
English Bull dog^b^	0	224 (4.3%)	
Chihuahua^b^	0	145 (2.8%)	
Labrador Retriever^a^	0	168 (3.2%)	
Pug^a^	0	135 (2.6%)	
Presa Canario^c^	0	107 (2.1%)	
Boxer^b^	0	99 (1.9%)	
Schnauzer group^a^, ^d^	0	81 (1.6%)	
Dalmata^b^	0	78 (1.5%)	
Bull terrier^b^, ^e^	0	74 (1.4%)	
Beagle	0	71 (1.4%)	
Podenco Canario^c^	0	56 (1.1%)	
Rottweiler^b^	0	55 (1.1%)	
English Staffordshire	0	53 (1.0%)	
Others (27 breeds)	0	551 (10.6%)	
Total	29 diabetic	5,184 non‐diabetic	

Note

Only breeds with more than 52 dogs (1% of the population) are represented.

Abbreviations: CI, Confidence IntervalOR, Odds Ratio.

^a^ Breeds previously reported to be at high risk.

^b^ Breeds previously reported to be at low risk for diabetes.

^c^ Local breeds not previously studied.

^d^ Schnauzer = giant (4), standard (62) and miniature (15) grouped.

^e^ Bull terrier = Standard (68) and miniature (6) grouped.

* (*p* <.05).

Regarding seasonality of the diagnosis for new cDM cases, no significant differences were observed among the different months [2.5 (1–4) cases per month, *p* =.457)] and seasons [6 (6–7) cases per season, *p* =.392].

Diabetes was clinically classified as diabetes secondary to dioestrus (DSD) (16 cases; 55.17%), idiopathic/immune‐mediated (5 cases; 17.24%), diabetes secondary to pancreatitis (DSP) (4 cases; 13.79%), iatrogenic (3 cases; 10.35%) and diabetes secondary to hypercortisolism (DSH) (1 case; 3.45%). The most commonly associated disorders were dermatologic diseases (31%) and pyometra (10%), although other conditions such as renal disease (6.9%) were also seen. In two dogs with recurrent atopic dermatitis, cyclosporin A and glucocorticoid treatment were suspected to be associated with the development of diabetes and, in another case, exogenous administration of progesterone was suspected to be the cause. The mean age of the dogs with the different types of diabetes were 8.85 years (4.65–12) for DSD, 9.6 years (3.5–12.1) for idiopathic/immune‐mediated, 13.25 years (11–13.6) for DSP, 7.75 (5.75–9.7) for iatrogenic and 8 for DSH.

Twenty five dogs received insulin treatment prior to sampling [porcine insulin (*n* = 23), NPH insulin (*n* = 1) and detemir (*n* = 1); treatment duration 35 (1–1155) days], and serum was available from 19 of them. Anti‐insulin antibodies were negative in all of these samples. Of the cases clinically suspected to be idiopathic/immune‐mediated (*n* = 5), autoantibody reactivity was confirmed in two cases (one to canine GAD65 and one to canine IA‐2). Although previously described high‐risk DLA‐alleles were not identified (Table 3), some of the DLA alleles found were common to several dogs.

**TABLE 3:** Characterization of the five cases suspected to have autoimmune diabetes

Breed	Sex	Age at diagnosis (years)	Age at sampling (years)	DLA alleles			Auto‐antibody reactivity	
				DRB1	DQA1	DQB1	GAD65	IA2
Griffon	MN	8.6	8.8	015/015	006/006	049/049	—	—
West Highland White Terrier	M	3.17	5.5	001/001	009/009	00101/03001	+	—
Miniature Poodle	FN	10.4	10.9	01501/01302 or 01503/01302	00101/02201	02601/00201	—	—
Poodle	M	12.1	12.1	01501/01301 or 01501/01302	009/009	001/001	—	+
Cross‐breed	M	7.8	8	001/001	006/006	049/049	—	—

Note

Abbreviations: FN, female, neutered; M, male; MN, male, neutered.

**FIGURE 1:**
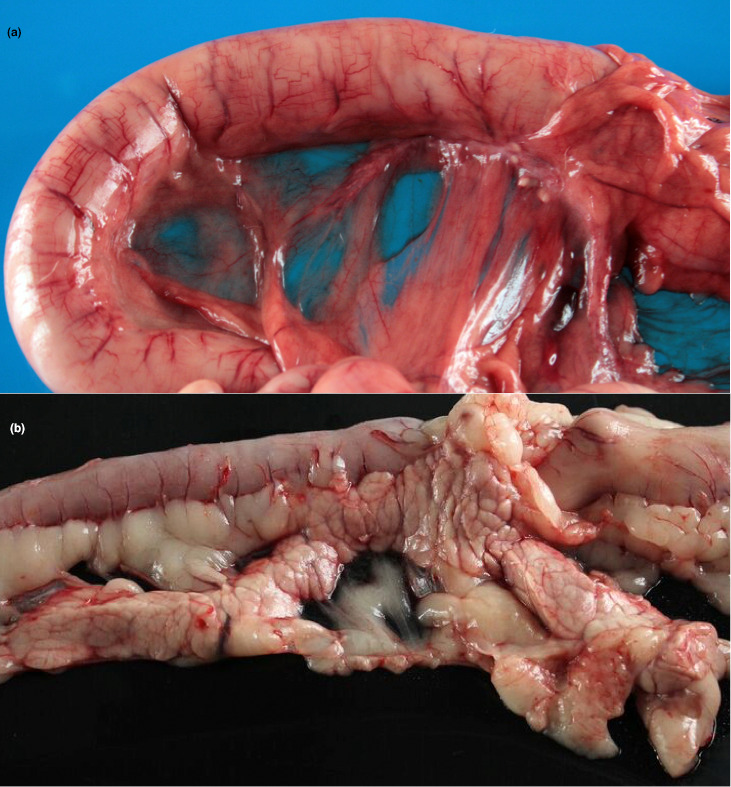
Pancreatic atrophy in a dog with diabetes mellitus: Post‐mortem examination of three diabetic dogs revealed pancreatic atrophy. In these images two diabetic pancreases are shown, the first with evident (a) and the second without apparent atrophy (b). Note the duodenum (left and superior flexure) and the residual pancreatic tissue embedded in the omentum, hardly detectable macroscopically (a). In b, both the duodenum and the pancreas are easily recognized

**FIGURE 2:**
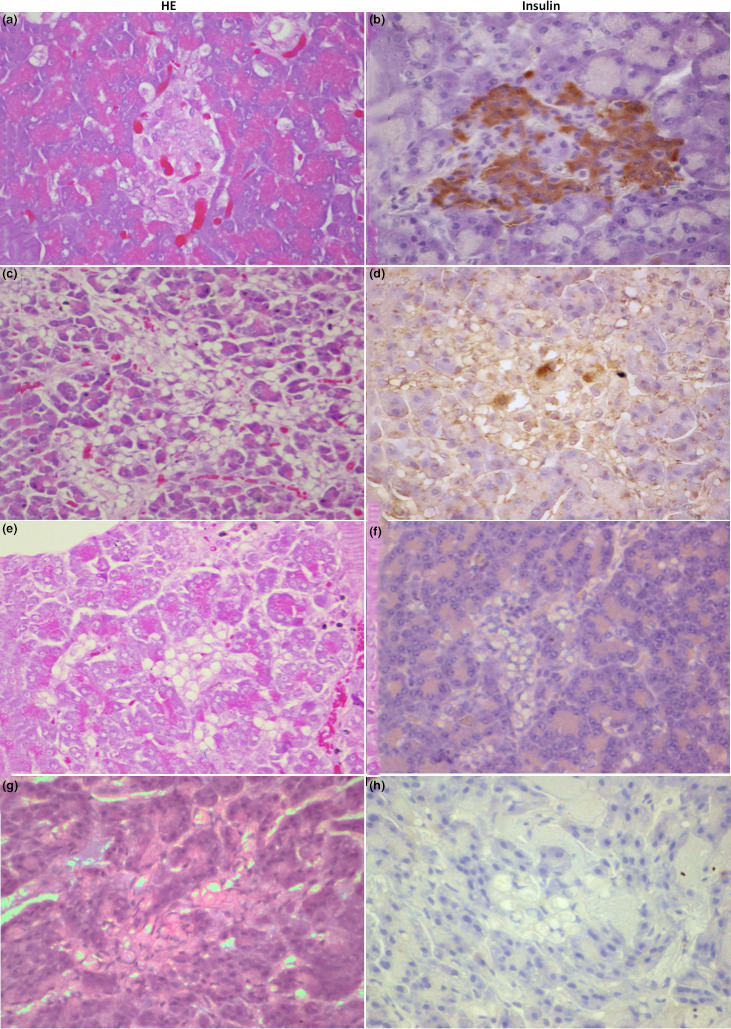
Histopathological evaluation: Haematoxilin‐Eosin (HE) and Insulin staining (Insulin) of non‐diabetic (a, b) and diabetic pancreases (c–h). No lesions are identified in the non‐diabetic tissues (a). Pancreatic islets can be easily recognized by the abundant, insulin‐positive (brown) staining (b). All diabetic pancreases showed a reduction in islet number and size, and when detected, vacuolation of islets cells and ductal epithelium was evident (c, e, g). Insulin positive‐cells were scant (D), with weak or absent immunostaining (f, h). An insulin‐staining grade was identified among all diabetic dogs, as is the case of D, a recently diagnosed (one week) dog with cDM secondary to hyperadrenocorticism, F, a pancreas from a dog with sustained hyperadrenocorticism, and H, a recently‐diagnosed immunemediated/idiopathic case (1 day)

Regarding pancreatic samples, pancreases from ten diabetic dogs were collected between 2009 and 2015 and were examined and compared with those of four non‐diabetic dogs. The most important findings are summarized in Table 4. Macroscopically, three of the diabetic dogs showed evidence of pancreatic atrophy (Figure 1a). Histopathologically, a reduction in pancreatic islet number and size was seen in four of the diabetic dogs and, when present, islets were sparse and scattered. In the other cases, there were apparently no differences in the quantity of islets compared to non‐diabetic pancreases. Vacuolation of islet cells and ductal epithelium was evident throughout all the tissue sections examined in most of the cases **(**Figure 2c–f).

**TABLE 4:** Histopathological features found in the diabetic pancreases analysed

Breed	Sex	Age (y)	Type of diabetes	Time since diagnosis	Pancreatic atrophy	Endocrine Pancreas		Exocrine Pancreas		Interlobular septa	Peripancreatic adipose tissue	
						*N* of islet cells	Vacuola‐tion	Inflam‐matory infiltrate	Necrosis	Inflammatory infiltrate	Inflamma‐tory infiltrate	Necrosis
Dachshund	F	13.25	DSP	39 days	—	Not observed	—	+	+	+	+	+
Siberian Husky	F	13.25	DSD	3.5 years	—	Not observed	—	—	—	—	+	—
Poodle	FN (at sampling)	12.7	DSD	2.1 years	—	Not observed	—	—	—	—	‐	—
German shepherd	F	9.25	DSD	7 days	—	Sparse	+	—	—	—	‐	—
Mixed	FN	15	DSD	3 years	+	Normal^a^	+	—	—	—	+	+
Mixed Poodle	F	12	DSH and DSP	45 days	—	Normal^a^	+	—	—	+	—	—
Yorkshire terrier	F	12	DSH and DSP	7 days	+	Normal^a^	+	+	+	+	—	—
Yorkshire terrier	F	5	DSH and DSP	NA	—	Normal^a^	+	+	—	—	—	—
Golden retriever	F	15	II	1 days	—	Normal^a^, ^b^	+	—	—	—	—	—
Yorkshire terrier	M	14	DSH	NA	+	Normal^a^	+	—	—	—	—	—

Note

Abbreviations: d, days; DSD, diabetes secondary to diestrus; DSH, secondary to hyperadrenocorticism; DSP, diabetes secondary to pancreatitis; F, Female; FN, female, neutered; II, idiopathic‐immunemediated, NA, not available; M, male; m, months; y, years.

^a^ Apparently no different quantity of islets compared to a non‐diabetic pancreas.

^b^ No insulin staining was detected even islets cells were apparently normal.

In two cDM cases, interlobular connective tissue was moderately expanded and multifocally, densely infiltrated by neutrophils and lesser macrophages, lymphocytes and plasma cells, with fibrin, oedema and necrotic debris. Occasionally, this inflammatory infiltrate extended into the adjacent exocrine parenchyma, with few and discrete areas of parenchymal lytic necrosis.

A single case (see Table 4: *Siberian husky*), showed mild to moderate multifocal necrosis and saponification of the peripancreatic adipose tissue, moderate infiltration of neutrophils and macrophages, rare multinucleated giant cells, and fewer lymphocytes and plasma cells.

In all non‐diabetic, control dogs, pancreatic islets were more abundant, evident and easily recognizable. No endocrine lesions were found in control dogs (Figure 2). However, in one case (non‐diabetic control, *Siberian husky*), multifocal, mild to moderate perivascular and periductal lymphocytes and plasma cell infiltration was observed. The interlobular septa, pancreatic parenchyma and peripancreatic adipose tissue were also affected, multifocally, by a small to moderate number of lymphocytes and plasma cells and scattered neutrophils and macrophages. In all controls, there was strong diffuse cytoplasmic insulin‐immunolabelling in the pancreatic islets. In the diabetic cases, insulin positive‐cells were scant, with weak or even absent immunolabelling. A semi‐quantitative insulin‐staining gradient was identified among all diabetic dogs. Time since diagnosis and type of diabetes seemed to be related to the number of insulin positive cells and respective insulin content (Figure 2).

### 1.3 DISCUSSION

The present study shows similar prevalence and incidence of diabetes as those reported in other regions. Most cases were non‐neutered females at diagnosis, with a clinical history supporting DSD. Of the five dogs classified as idiopathic/immune‐mediated by their clinical presentation, two showed evidence of autoantibody reactivity, and new DLA alleles were identified.

The present study was performed in a reference Teaching Hospital that attends a population that has not been previously studied for cDM. All the hospital clinical records were reviewed for the described period to identify the cases of diabetes.

Age at diagnosis, prevalence and incidence were similar to those reported in previous studies performed in other populations (Davison et al., 2005; Fall et al., 2007; Fracassi et al., 2004; Mattin et al., 2014). However, a high frequency of DSD was seen, which could be explained by the high proportion of entire females found in our canine population, compared with others (Fracassi et al., 2004; Pöppl et al., 2017), where elective neutering is more common. Indeed, these results are in agreement with those found in geographical regions where neutering is less popular (Ahlgren et al., 2014; Pöppl et al., 2017). Moreover dioestrus has been identified as a major risk factor in the development of cDM (Pöppl et al., 2017). This reflects the importance of sterilization in females to prevent cDM, and to potentially revert its progression at early stages as previously stated (Pöppl et al., 2013).

In a recent population‐based study (Mattin et al., 2014), neutered males were at higher risk of developing diabetes compared to intact males, in contrast with the present study, where more than 80% of the diabetic males were intact.

Body weight and body condition score were not registered in most cases, and weight loss was frequent at diagnosis, which made it difficult to evaluate their distribution among the different types of diabetes. Likewise, the age distribution could not be assessed, either.

Compared with previous studies, the breed distribution and susceptibility to diabetes was similar for some breeds, but different for others. It is worthwhile to point out that the profile of dog breeds in a population varies considerably and that the genetic factors involved in determining susceptibility to cDM are likely to be population‐specific (Davison et al., 2005; Fall et al., 2007; Fracassi et al., 2004; Gale, 2005; Guptill et al., 2003; Mattin et al., 2014). For some high‐risk breeds, the present study shows even higher risks than previously described, suggesting that geographical isolation and inbreeding could play a role (Fracassi et al., 2004; Gale, 2005; Guptill et al., 2003; Mattin et al., 2014). The Andalusian wine‐cellar rat‐hunting dog (similar to the Jack Russel terrier) was identified as a high‐risk breed in the Canary Islands. Other breeds that are typically at high risk for diabetes according to previous studies (Samoyedo, schnauzer, pug, English setter) seem to be at low risk in our population, although they are not particularly prevalent in the dog population in this region. Furthermore, breeds that have been reported to be relatively less susceptible to cDM were identified as high‐risk in the present study (German shepherd and cocker spaniel) (Davison et al., 2005; Fracassi et al., 2004; Gale, 2005; Marmor et al., 1982; Yoon et al., 2020). In addition, five local, previously unstudied breeds did not show any evidence of the disease: Presa Canario, Podenco Canario, Bardino Majorero, Pastor Garafiano and Spanish water‐dog, although only a few dogs were available of the latter breed.

No seasonal patterns were found for the diagnosis in the present study, as has been demonstrated for cDM and human T1D in some (Ardicli et al., 2014; Atkins & MacDonald, 1987; Davison et al., 2005; Hanberger et al., 2014; Hess et al., 2000; Mattin et al., 2014; Moltchanova et al., 2009; Pattersonet al., 2009; Samuelsson et al., 2013), but not all previous reports (Guptill et al., 2003; Jensen et al., 2015), including human data from the Canary Islands (Domínguez, 2000; Novoa‐Medina et al., 2006–2013.). Although spring peaks in diagnosed cases are thought to be related to increased oestral activity in entire females, in the context of onset of DSD (Fall et al., 2007), the present study does not support this idea.

Autoantibodies against GAD65 and/or IA2 in dogs suspected of having immune‐mediated/idiopathic cDM were only identified in two out of five cases. Different reasons could account for the lack of autoantibody reactivity, such as the prolonged period between diagnosis and blood sampling, although the GAD65 autoantibody positive dog was sampled 2.4 years after diagnosis. Furthermore, other antibodies, such as those against pro‐insulin or zinc transporter 8, were not assessed in the present study and have been shown to be positive in other studies (Ahlgren et al., 2014; Davison et al., 2011; Merger et al., 2013). Indeed, it is possible that additional autoimmune targets involved in cDM are still to be discovered. The absence of anti‐insulin reactivity in all the insulin‐treated dogs evaluated, is in agreement with previous studies and could be explained by the homology between canine and porcine insulin leading to immunological tolerance (Davison et al., 2003).

The DLA haplotypes and genotypes observed in this population have, to our knowledge, not been described before and, consequently, have not been previously associated with cDM (Kennedy et al., 2006). They might be population‐specific, but since no other local non‐diabetic or diabetic dogs were genotyped, no further conclusions can be drawn at this point.

Cyclosporine A and glucocorticoids can cause insulin resistance and hyperglycaemia (Kovalik et al., 2011; Murray et al., 2009). In the same way, progesterone therapy can induce iatrogenic DSD (Selman et al., 1994). In the cases described in this report, all these treatments were administered to those classified as iatrogenic, and maybe the repeated administrations of these therapies in a pre‐diabetic situation and/or in high‐risk breeds, could trigger the development of diabetes.

A reduction in the number of islets and β‐cells, lymphocytic infiltration, insulitis, pancreatic inflammation and β‐cell vacuolation are the most pertinent pathological findings in diabetic dogs, although there is a degree of heterogeneity and discrepancies among studies (Alejandro et al., 1988; Atkins et al., 1979, 1988; Gepts, 1965; Gepts & Toussaint, 1967; Gilor et al., 2016; Jouvion et al., 2006; Pöppl et al., 2017; Shields et al., 2015). In the present report, a high proportion of diabetic pancreases showed a substantial reduction in the number of islets and β‐cell mass, as opposed to control pancreases, where islets were numerous and mostly constituted by β‐cells, consistent with previous findings and the commonly proposed pathogenesis with human LADA (Catchpole et al., 2005; Shields et al., 2015). Time since diagnosis and the cause of cDM could also have an effect on the level of β‐cell loss, which is more evident with longer duration of the disease. In this sense, although our sample size is small, it seems that cDM secondary to hyperadrenocorticism and DSD, where there is a predominance of insulin resistance, showed slower progression of β‐cell loss, compared to the drastic loss in immune‐mediated or pancreatitis‐associated cases. This would be explained by the glucotoxicity effect associated to the sustained hyperglycaemia, which promotes a slower, and reversible at early‐stages, progression of β‐cell loss (Habib‐Ur‐Rehman et al., 2020; Link et al., 2013).

The most important limitation of the present study is probably its small sample size, maybe not representative of the whole Canarian canine population. This could have affected certain evaluations, like the distribution of age and weight according to type of diabetes. The lack of local control groups for DLA and autoantibody measurements, and the impossibility to perform all of the evaluations (histopathology, genetic and serum analysis) in the same diabetic cases, also limits some of the conclusions. Nevertheless, despite these limitations, the results obtained contribute to the overall knowledge on canine diabetes, from a yet undescribed population. To the best of our knowledge, this is the first study characterizing cDM in Spain.

### 1.4 CONCLUSIONS

In conclusion, in the Canary Islands, where neutering is not standard practice, DSD is the most frequent subtype of diabetes. Despite the limitations associated with small sample size, our findings support the marked influence of breed and possible genetic factors on the susceptibility to cDM, as well as other factors such as neutering status and administration of insulin antagonist drugs. Indeed, heterogeneity in cDM is probably comparable to that of human diabetes.

Additional population‐based studies in different geographical regions are still necessary to assess the heterogeneous nature of cDM.

## CONFLICTS OF INTEREST

The authors declare no conflicts of interest.

## AUTHOR CONTRIBUTION

**Yeray Brito‐Casillas:** Conceptualization; Data curation; Funding acquisition; Investigation; Methodology; Resources; Software; Writing‐original draft. **Carlos Melian:** Data curation; Formal analysis; Investigation; Methodology; Supervision; Writing‐review & editing. **Angela Holder:** Investigation; Methodology; Supervision; Writing‐review & editing. **Julia C. Wiebe:** Data curation; Funding acquisition; Investigation. **Ana Navarro:** Investigation; Methodology; Software; Writing‐review & editing. **Óscar Quesada‐Canales:** Investigation; Methodology; Supervision; Writing‐original draft. **Ana Expósito‐Montesdeoca:** Investigation; Methodology. **Brian Catchpole:** Conceptualization; Supervision; Writing‐review & editing. **Ana M. Wägner:** Conceptualization; Formal analysis; Investigation; Project administration; Supervision; Writing‐review & editing.

## ETHICAL STATEMENT

This study was approved by the University's Animal Welfare Ethics Committee (Comité Ético de Bienestar Animal, CEBA‐ULPGC), and the owners gave informed consent before the samples were obtained, to include their dogs in the study.

### PEER REVIEW

The peer review history for this article is available at https://publons.com/publon/10.1002/vms3.452.

## Data Availability

The datasets used and/or analysed during the current study are available from the corresponding author on reasonable request.
